# Clinical management of bleeding manifestations in a family with the thrombomodulin C1611>A (p.Cys537Stop) mutation

**DOI:** 10.1016/j.rpth.2025.102678

**Published:** 2025-01-16

**Authors:** Serge Pierre-Louis, Johalene Rabout, Octavio Labrada, Fatima Radouani, Emeline Chonville, Beatrice Ferrey, Olivier Pierre-Louis, Yesim Dargaud

**Affiliations:** 1 Centre de Ressources et de Compétences des Maladies Hémorragiques Constitutionnelles, CHU de Martinique, Hôpital Pierre Zobda Quitman, Fort-de-France, Martinique; 2 Centre Hospitalier Universitaire de la Martinique, Hôpital Pierre Zobda Quitman, Service de Chirurgie Orthopédique, Fort-de-France, Martinique; 3 UR5_3 Pathologies cardiaques, toxicités environnementales et envenimations, Université des Antilles, Hôpital Pierre Zobda Quitman, Fort-de-France, Martinique; 4 Centre de Référence de l’Hémophilie, Unité d'Hémostase Clinique, Hôpital Cardiologique Louis Pradel, Université Lyon I, Lyon, France

**Keywords:** bleeding, C1611>A, p.Cys537Stop, platelet transfusion, recombinant activated factor VII (rFVIIa), thrombomodulin (TM)

## Abstract

**Background:**

The bleeding disorder described here is due to a heterozygous autosomal dominant C1611>A variant in the thrombomodulin (TM) gene that significantly elevates plasma TM levels, which enhances the activation of protein C. This activation inhibits factors VIIIa and Va, reducing thrombin generation and potentially leading to severe hemorrhagic manifestations.

**Key Clinical Question:**

What is the bleeding profile of patients with this rare condition? What are the most frequent clinical signs, and how can they be treated?

**Clinical Approach:**

We present a case study of an index patient with the *TM* C1611>A variant and his 20 family members. We detail the hemostatic strategies employed during various bleeding episodes and surgical procedures.

**Conclusion:**

Sharing clinical experiences is crucial for hematologists managing similar cases, as it provides valuable insights into effective treatment strategies.

## Essentials

• Thrombomodulin (TM) C1611>A; p.Cys537Stop variant is a very rare condition.
• Patients with this TM variant experience trauma/surgery-related bleeding.
• Clinical experience on TM C1611>A is scarce.
• This work describes the largest series of patients and treatment strategy.

## 1 Introduction

Thrombomodulin (TM) is a glycoprotein that is expressed in human endothelial cells and a number of other cell types, including hematopoietic progenitors, dendritic cells, and monocytes [1]. It plays a critical role in regulating coagulation by accelerating thrombin-mediated protein C activation over 1000-fold compared with thrombin (FIIa) alone. When complexed with TM, thrombin’s substrate specificity shifts from procoagulant to anticoagulant [2]. Additionally, TM plays a role in innate immunity, inflammation, and cell growth, and it is a marker of endothelial injury in conditions such as diabetes, disseminated intravascular coagulation, atherosclerosis, and cancer [3].

Human TM is a 557-amino acid transmembrane glycoprotein encoded by the intronless *THBD* gene on chromosome 20p11.21 [4]. Several *THBD* gene polymorphisms, including promoter variants, have been identified, but their impact on soluble TM levels is inconsistent and needs further investigation [[5], [6], [7]].

**Figure 1:**
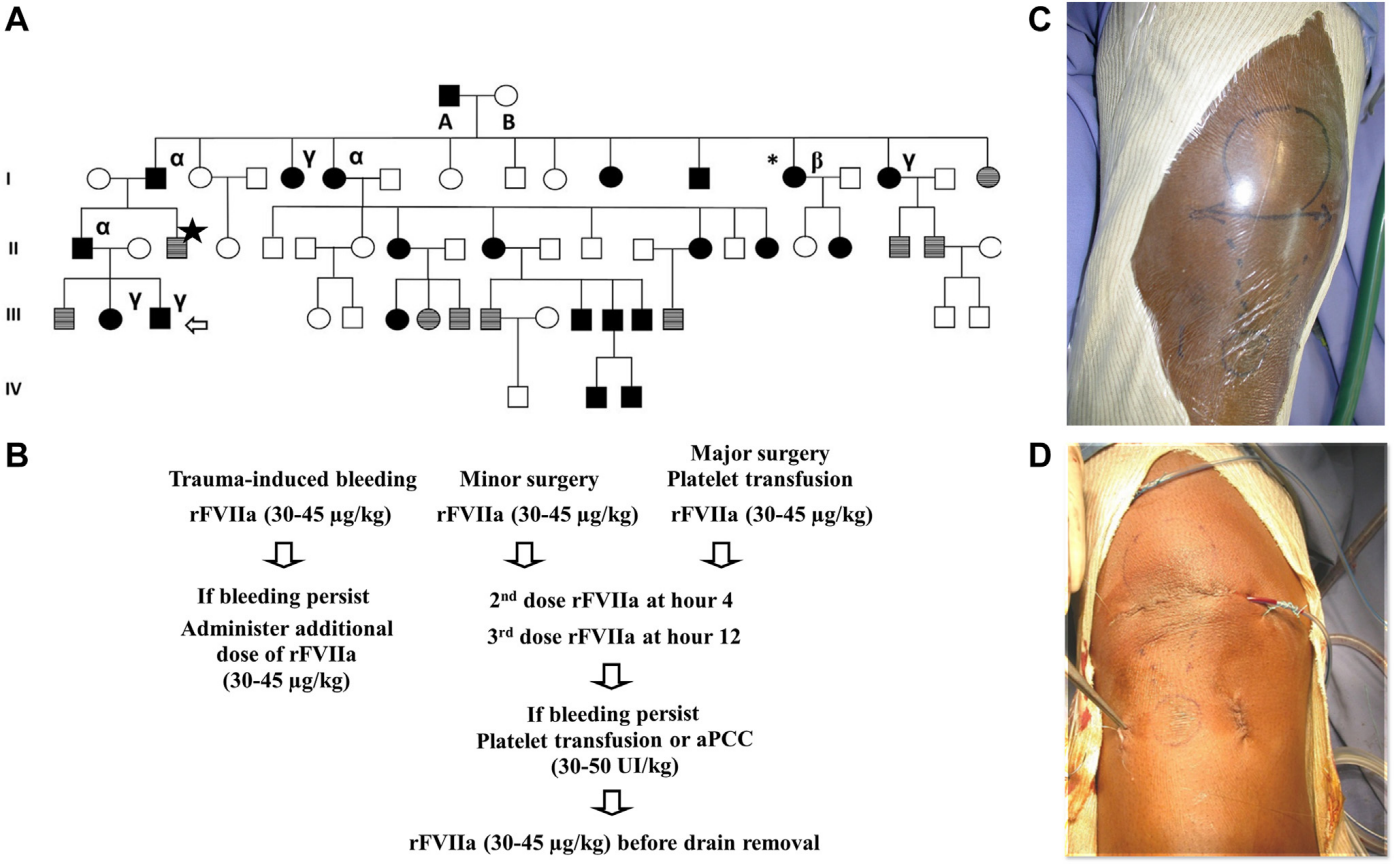
(A) Family tree of the patients with thrombomodulin ™ C1611>A and their soluble TM levels. Black circles and squares: patients with objectively diagnosed *TM* variant and personal history of bleeding. Gray circles and squares: asymptomatic patients with normal soluble TM levels (2.9-7.6 ng/mL). White circles and squares: clinically asymptomatic patients with no laboratory testing. (B) Proposed therapeutic strategy for the treatment of bleeding episodes in patients with the *TM* C1611>A variant. Left leg hematoma (C) before and (D) after surgery. *aPCC*, activated prothrombin complex concentrates; rFVIIa, recombinant activated factor VII.

A rare *TM* mutation (c.1611C>A) that codes for a change from cysteine 537 to a premature stop codon (p.Cys537Stop) associated with the truncation and shedding of variant TM from the endothelial surface into the blood was previously reported [[8], [9], [10]]. Very high levels of soluble TM promote systemic protein C activation and inhibition of FIIa generation [9]. We describe here the clinical management of a large family with the p.Cys537Stop mutation living on the French Caribbean island of Martinique (Figure 1A). The authors obtained informed consent of the patient for this case report and followed the CARE (CAse REports) guidelines.

## 2 Case Report

The index patient, a 26-year-old male, was injured in a road accident in 2005, resulting in a large hematoma in his left leg due to a ruptured patellar tendon (Figure 1C, D). His medical history is otherwise unremarkable. However, a detailed family history reveals a pattern of trauma- or surgery-related hemorrhage affecting several relatives over several generations. His maternal aunt suffered severe bleeding and hemorrhagic shock after pelvic surgery for an ovarian cyst in Lyon in 1998 [9], controlled by platelet transfusions. Her preoperative laboratory evaluation of hemostasis was completely normal. However, the prothrombin (FII) consumption test was significantly abnormal and FIIa generation was dramatically reduced [9]. At that time, the *TM* p.Cys537Stop variant had not yet been identified or described in the literature yet.

Based on the successful platelet transfusion of the aunt treated in Lyon, the patient underwent patellar tendon reconstruction surgery following a platelet transfusion at a standard dose of 0.5 × 10^11^ platelets per 10 kg of body weight. Postoperatively, the patient received daily platelet transfusions for 3 days without bleeding complications. The patient experienced fever and chills as a reaction to the platelet transfusions. Operative blood loss was approximately 1000 mL, resulting in a 3-point decrease in hemoglobin levels. Four months later, the patient sustained a new trauma to the same knee, resulting in a large hematoma involving the quadriceps, knee, and calf, with hemoglobin dropping to 6 g/dL.

This required transfusion of 4 units of allogeneic red blood cells and 9 units of platelet concentrates. The patient experienced significant shivering and hyperthermia, which improved with high-dose corticosteroids. Laboratory analyses revealed the absence of circulating antiplatelet alloantibodies directed against glycoproteins Ia-IIa (GPIa-IIa), IIb-IIIa (GPIIb-IIIa), and Ib-IX (GPIb-IX). Additionally, no anti-HLA class I or II antibodies were detected. On postoperative day 10, despite hematoma regression, the patient developed sepsis from the infected hematoma, unresponsive to broad-spectrum antibiotic therapy. Urgent surgical evacuation of the hematoma was required. Given the severity of the side effects observed with the recent platelet transfusions and the patient’s deteriorating general condition, recombinant activated factor VII (rFVIIa, NovoSeven, Novo Nordisk) was administered off-label as an alternative treatment. The hematoma was successfully evacuated without excessive bleeding using rFVIIa at a dose of 30 μg/kg, administered preoperatively and then every 4 hours for 12 hours. In this patient, who had no personal history of bleeding, no identified bleeding-related diagnosis, and normal coagulation and primary hemostasis test results, we opted for a low dose of rFVIIa to manage bleeding. This decision was carefully made after weighing the potential risk of thrombosis in this clinical context. However, on postoperative day 10, during drain removal, which was performed without procoagulant treatment, significant bleeding occurred, resulting in a significant drop in hemoglobin. This required transfusion of 2 units of red blood cells and an additional dose of rFVIIa at 30 μg/kg.

One month after reconstructive patellar tendon surgery, a new operation to remove the medial gastrocnemius muscle through a long incision extending from the quadriceps to the calf was successfully performed with rFVIIa 30 μg/kg administered immediately before surgery and at 4 and 12 hours postoperatively. The drain was removed after a single dose of rFVIIa. Fifteen days later, a large skin graft from the patient’s buttocks was placed over the wound with 2 doses of rFVIIa 30 μg/kg administered preoperatively and 4 hours postoperatively. The graft site was completely healed by day 30.

Faced with this unusual clinical case, we conducted a comprehensive evaluation of the patient’s entire family living on the island of Martinique. We identified 21 members, including 9 males and 12 females, who exhibited clinical signs of hemorrhage. The youngest diagnosed family member was 4 years old, while the oldest was an 80-year-old woman. None of the patients had a history of spontaneous bleeding. The most common bleeding manifestations were hematomas and prolonged bleeding after dental extractions, which could last for more than 15 days. No cases of heavy menstrual bleeding were reported unless associated with underlying uterine pathology such as fibroids or uterine polyps. Pregnancies and deliveries were normal in the majority of cases. The few cases of delivery-related bleeding were secondary to vaginal or uterine injury or episiotomy. However, all cesarean sections were markedly hemorrhagic. We did not observe any cases of intracranial hemorrhage or hemarthrosis, even after significant trauma such as a tibial plateau fracture associated with a significant hematoma. A 20-year-old patient presented with a calcified hematoma on the posterolateral aspect of the right knee, which was attributed to repeated sports-related trauma.

Laboratory evaluations of all family members showed normal results, except for a significantly increased FII consumption test in some individuals compared to the control. Interestingly, family members with no history of bleeding had normal FII consumption results despite previous surgery. Residual FII levels were measured in serum samples 5 and 24 hours after coagulation of the patient’s blood at 37 °C using the determination antigen by immunological method. The FIIa generation assay was not available in Martinique at that time. A plasma bank and DNA library were established.

Following the successful management of the index patient’s hematoma with a low dose of rFVIIa, subsequent trauma-induced bleeding episodes in this family—including hematomas and injuries to the gums, lips, mouth, and tongue—as well as minor surgical procedures, such as dental extractions and carpal tunnel surgery, have been effectively treated with rFVIIa administered at a dose of 30 μg/kg. In surgical settings, a second consolidation dose was administered 4 hours later. Joint infiltrations as well as vaccinations and other intramuscular injections were not associated with bleeding and did not require preventive treatment. There is no experience with lumbar puncture in this family.

Following the identification of the *TM* C1611>A variant, which follows an autosomal dominant pattern of inheritance, in 2 independent patients in late 2014 and early 2015 [8,9], including our patient’s aunt treated at the Lyon Hemophilia Center [9], we sent plasma and DNA samples from family members to the reference center in Lyon for further analysis. Laboratory tests confirmed the diagnosis, revealing 10 to 50 times higher levels of soluble TM in all family members with a history of bleeding. Notably, the level of circulating TM was not correlated with the frequency or severity of bleeding symptoms.

## 3 Discussion

Bleeding symptoms associated with the *TM* C1611>A variant are never spontaneous but are induced by trauma or surgery. They are delayed and disproportionate to the cause, do not stop spontaneously, and may recur if inadequately treated. There is significant interindividual variability of bleeding risk depending on plasma levels of TM. Our clinical experience indicates that in patients with the *TM* C1611>A variant, no invasive procedure, even simple ones such as drain removal, should be performed without prophylactic hemostatic treatment. The therapeutic strategy aims to increase FIIa generation to achieve hemostasis without increasing thrombotic risk. Nonspecific procoagulant treatment options include rFVIIa, activated FII complex concentrate, platelet concentrates, and tranexamic acid [11]. In our experience, low-dose rFVIIa at 30 μg/kg was associated with excellent to good clinical efficacy (Figure 1B). In surgical situations, patients received an additional dose in the fourth hour. FII consumption test results do not normalize with rFVIIa and laboratory monitoring was not performed. However, the FIIa generation assay, a global hemostasis test that integrates the effects of all procoagulant and anticoagulant proteins, can theoretically be used as a monitoring test, as has been reported in hemophilia patients with inhibitors treated with rFVIIa [12,13].

Given the large number of patients in Martinique, we developed a specific therapeutic education program for this rare disease [14]. Insights from consultations helped us refine the program and establish a therapeutic approach. Data collection was crucial in defining the bleeding profile. Group discussions with 4 to 6 patients and their families fostered a trustful atmosphere, essential for sharing information and experiences. Motivational interviewing has been key in managing patients, often diagnosed late in life. This method helped address emotions such as fear and sadness and was favored by the healthcare team. We considered patients’ life experiences and coping skills, recognizing that late-diagnosed patients had developed their own self-care practices. Through empathetic listening and positive argumentation, we helped patients change risky behaviors and adhere to the therapeutic plan.

We report the largest series of patients with the *TM* C1611>A; p.Cys537Stop variant. The isolation of Martinique, with its higher risk of consanguinity, may explain the prevalence of this autosomal dominant disorder in our center. This rare bleeding disorder is often poorly recognized by healthcare professionals, and clinical experience is limited. The diagnostic delay has affected patient care, but our family study has enabled earlier diagnosis in younger members. We hope that sharing our experience will assist other patients awaiting diagnosis and appropriate treatment.
